# Pulmonary Targeting of Levofloxacin Using Microsphere-Based Dry Powder Inhalation

**DOI:** 10.3390/ph15050560

**Published:** 2022-04-30

**Authors:** Turki Al Hagbani, Bhavya Vishwa, Amr S. Abu Lila, Hadil Faris Alotaibi, El-Sayed Khafagy, Afrasim Moin, Devegowda V. Gowda

**Affiliations:** 1 Department of Pharmaceutics, College of Pharmacy, University of Hail, Hail 81442, Saudi Arabia; t.alhagbani@uoh.edu.sa (T.A.H.); a.abulila@uoh.edu.sa (A.S.A.L.); 2 Department of Pharmaceutics, JSS College of Pharmacy, Mysuru 570015, India; bhavyavishwa@gmail.com; 3 Department of Pharmaceutics and Industrial Pharmacy, Faculty of Pharmacy, Zagazig University, Zagazig 44519, Egypt; 4 Department of Pharmaceutical Sciences, College of Pharmacy, Princess Nourah bint AbdulRahman University, Riyadh 11671, Saudi Arabia; hfalotaibi@pnu.edu.sa; 5 Department of Pharmaceutics, College of Pharmacy, Prince Sattam bin Abdulaziz University, Al-kharj 11942, Saudi Arabia; e.khafagy@psau.edu.sa; 6 Department of Pharmaceutics and Industrial Pharmacy, Faculty of Pharmacy, Suez Canal University, Ismailia 41522, Egypt

**Keywords:** inhalable microspheres, levofloxacin, lung targeting, pulmonary drug delivery, tuberculosis

## Abstract

The objective of the current study was to develop poly (lactic-co-glycolic acid) (PLGA) microspheres loaded with the anti-tuberculosis (anti-TB) fluoroquinolone, Levofloxacin (LVX), in the form of dry powder inhalation (DPI). LVX-loaded microspheres were fabricated by solvent evaporation technique. Central Composite Design (CCD) was adopted to optimize the microspheres, with desired particle size, drug loading, and drug entrapment efficiency, for targeting alveolar macrophages via non-invasive pulmonary delivery. Structural characterization studies by differential scanning calorimetry (DSC), Fourier transform infrared (FTIR) spectroscopy, and X-ray diffraction analysis revealed the absence of any possible chemical interaction between the drug and the polymer used for the preparation of microspheres. In addition, the optimized drug-loaded microspheres exhibited desired average aerodynamic diameter of 2.13 ± 1.24 μm and fine particle fraction of 75.35 ± 1.42%, indicating good aerosolization properties. In vivo data demonstrated that LVX-loaded microspheres had superior lung accumulation, as evident by a two-fold increase in the area under the curve AUC_0–24h_, as compared with plain LVX. Furthermore, LVX-loaded microspheres prolonged drug residence time in the lung and maintained a relatively high drug concentration for a longer time, which contributed to a reduced leakage in the systemic circulation. In conclusion, inhalable LVX-loaded microspheres might represent a plausible delivery vehicle for targeting pulmonary tuberculosis via enhancing the therapeutic efficacy of LVX while minimizing its systemic off-target side effects.

## 1. Introduction

Tuberculosis (TB), the second leading infectious cause of death after COVID-19, is a major global health challenge. Tuberculosis is caused mainly by *Mycobacterium tuberculosis* bacilli [1]. The bacilli can go completely dormant after infecting humans without even showing noticeable symptoms for many years, or it can cause an active illness within few months of infection [2]. TB commonly affects the lungs (pulmonary tuberculosis), but it can affect other parts of the body (extra pulmonary TB) as well [3].

Generally, the standard first-line treatment regimen of tuberculosis is the six-month combination therapy of rifampicin, isoniazid, pyrazinamide, and ethambutol. Nevertheless, despite its efficacy against drug-susceptible tuberculosis, challenges such as poor patient adherence to the treatment and development of severe side effects could adversely affect the therapeutic outcomes of such treatment regimen [4,5]. Most importantly, the emerging of multi-drug resistance (MDR) is accompanied by a higher incidence of treatment failures and disease recurrence [6].

Recently, fluoroquinolones have been introduced as an essential component of the treatment regimens of multi-drug resistant TB (MDR-TB) [7]. They act by targeting DNA topoisomerase and gyrase enzymes in the bacteria and have shown the potential to reduce treatment duration in murine models of TB [8]. Levofloxacin and moxifloxacin are the two most potent fluoroquinolones currently in use as core agents in treatment regimens of MDR-TB [9]. Moxifloxacin was reported to exert a higher in vitro drug susceptibility than levofloxacin; however, upon long-term treatment, moxifloxacin could cause significant QT prolongation, increasing the risk of fatal arrhythmia. On the other hand, levofloxacin (LVX) administered at even higher doses than the therapeutic doses was well tolerated and only caused minimal QT prolongation. The better safety profile of LVX, compared to moxifloxacin [10], made it the preferred fluoroquinolones for combination with other agents that do prolong the QT interval, such as clofazimine, delamanid, or bedaquiline, which constitute the main core of novel TB treatment shortening regimens. Nevertheless, gastrointestinal symptoms, including nausea, vomiting, dyspepsia, and diarrhea, encountered upon oral administration of LVX may limit its use in many clinical settings.

The pulmonary route of administration has been traditionally used for drug administration to the respiratory tract for the treatment of various diseases, particularly those affecting the lungs. In addition, pulmonary drug delivery opens new avenues for drug administration to the lungs, both for localized and systemic therapy [11]. Compared to oral or parenteral routes of drug administration, the potential utility of the lungs as an entry for drugs relies on the exploitation of the unique features of lungs such as large surface area, highly permeable thin alveolar epithelium, high vascularization, and limited enzymatic activity, which collectively could enhance drug pharmacokinetics and, thereby, lead to high drug bioavailability [12,13]. Recently, many reports have emphasized the superior therapeutic outcomes of pulmonary administered LVX against various lung diseases, compared to orally administered drug [14,15,16].

Although carrier-free inhalation formulations are recommended due to their better toxicological profile, polymeric particulate-based pulmonary systems have demonstrated significant promise for improving drug bioavailability and therapeutic efficiency of the inhaled drug [17]. Among various natural and synthetic polymers, biodegradeable/biocompatible polymer carriers, such as poly (lactic co-glycolic) acid (PLGA) particles, represent an attractive option for inhalation therapies [18]. PLGA particles hold the potential to bestow higher physicochemical stability for the entrapped drug, permit sustained delivery of the inhaled drug to the lung and, when adequately engineered, it could ensure site-specific drug targeting [18,19,20].

The aim of this study was to formulate PLGA microspheres loaded with the anti-tuberculosis agent, LVX, for pulmonary delivery. The aerosolization performance of the formulated microspheres was evaluated in vitro. In addition, the in vivo fate of LVX-loaded microspheres was investigated post pulmonary administration. 

## 2. Results and Discussion

### 2.1. Preparation of LVX-Loaded Microspheres

#### 2.1.1. Central Composite Design (CCD)

A two-level, two-factor central composite design (CCD) was implemented for the formulation and optimization of LVX-loaded microspheres. CCD offers the advantage of creating a quadratic model for the response variables without the need of using a complete three-level factorial experiment. In this study, a total of 13 formulations (Table 1) were obtained by modifying two formulation parameters; drug concentration (X_1_) and polymer (PLGA) concentration (X_2_), and their effect on three formulation responses; namely, particle size (Y_1_), percentage drug loading (Y_2_) and percentage entrapment efficiency (%EE, Y_3_) was investigated. Multiple regression analyses were run on the responses set for optimizing drug formulation and second-order polynomial equations were derived from the selected model, based on regression coefficient values, as represented in the following three equations:

Y_1_ = +38.95 − 1.467 X_1_ − 2.361 X_2_ + 1.485 X_1_X_2_ − 2.339 X_1_^2^ + 0.653 X_2_^2^

Y_2_ = +79.912 + 0.404 X_1_ + 0.145 X_2_ + 1.205 X_1_X_2_ − 7.303 X_1_^2^ − 1.623 X_2_^2^

Y_3_ = +2.92 + 0.256 X_1_ − 0.326 X_2_ − 0.05 X_1_X_2_ + 0.127 X_1_^2^ + 0.227 X_2_^2^

**Figure 1:**
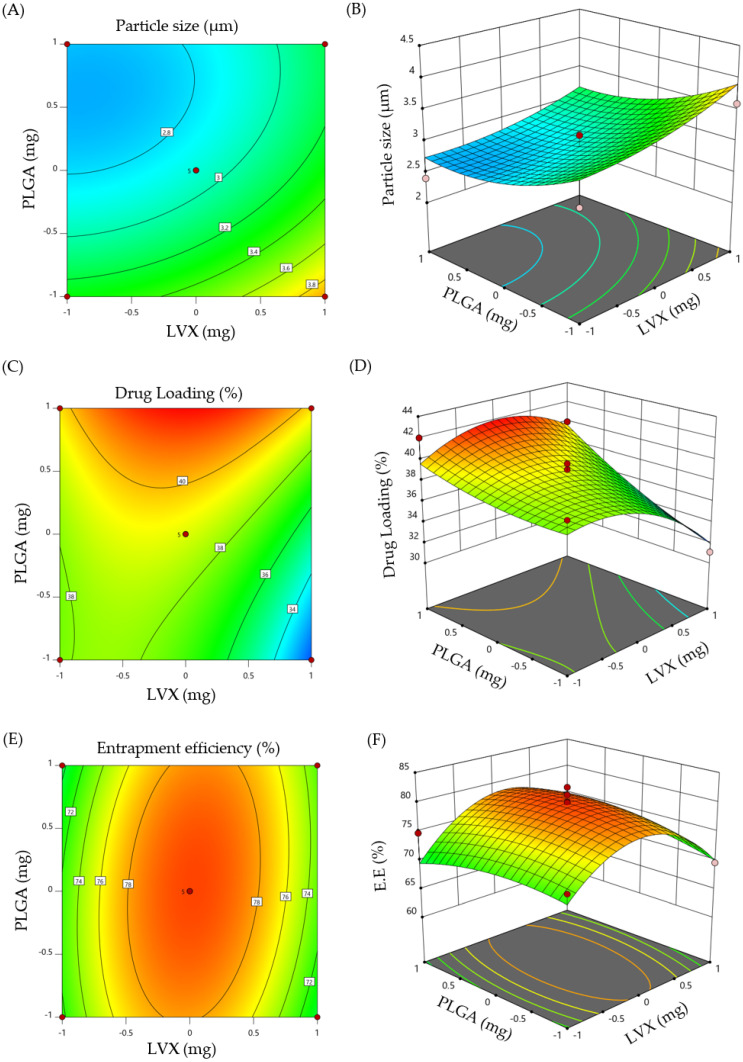
(**A**) Contour plot of particle size (Y_1_); (**B**) 3D surface plot for Y_1_; (**C**) Contour plot of drug loading (Y_2_); (**D**) 3D surface plot for Y_2_; (**E**) Contour plot of entrapment efficiency (Y_3_); and (**F**) 3D surface plot for Y_3_.

The significance and magnitude of the tested dependent variables on the independent responses was elucidated by response surface plot and three-dimensional (3D) plot (Figure 1). ANOVA test was used to test their significance. It was obvious that both polymer content and drug concentration exerted variable responses on the estimated formulation responses. A positive sign denotes a synergistic effect, whilst, a negative sign denotes an antagonistic effect of the factor on the selected response. 

#### 2.1.2. Optimization of LVX-Loaded Microspheres 

Desirability approach was applied to develop an optimized microsphere formula with desired responses, such as maximum drug loading, maximum encapsulation efficiency, and appropriate particle size within the aerosolable range (1–5 μm) [21]. The optimized LVX-loaded microsphere prepared under the aforementioned constrains was obtained at a polymer content of 10 mg and drug concentration of 7.8 mg. The particle size, % drug loading and % EE of the prepared optimized formula were 2.86 µm, 40.85% and 77.8%, respectively, which were close to predicted values (2.83 µm, 41.95% and 78.51%) of optimized formula obtained at a desirability value of 0.927.

### 2.2. Characterization of Optimized LVX-Loaded Microspheres

The physicochemical characteristics of microspheres for dry powder inhalation potentially affect the aerosolization behavior of microparticles upon inhalation. Accordingly, the physicochemical properties such as particle size, surface morphology, moisture content, flow property, and aerodynamic diameter of the optimized LVX-loaded microspheres were evaluated.

#### 2.2.1. Particle Size

Particles size is a key parameter that dictates lung deposition pattern of dry powder inhalers following pulmonary administration [21]. Generally, small particles with a particle size <1 μm are readily expelled before reaching lung tissue [22], whereas larger particles with size >5 μm are retained at upper respiratory tract and do not reach the deep lung tissue [22,23]. In addition, particle size plays a crucial role in targeting the particles to a specific lung region. Champion et al. [24] demonstrated that rifampicin-loaded PLGA microparticles with an average size of 1–6 μm were more efficient in targeting alveolar macrophages than larger particles. In the current study, optimized LVX-loaded microspheres showed an average particle size of 2.86 ± 0.26 μm (Figure S1), which is considered suitable for targeting LVX to alveolar macrophages; the major site of pulmonary tuberculosis infection.

#### 2.2.2. Particle Morphology

**Figure 2:**
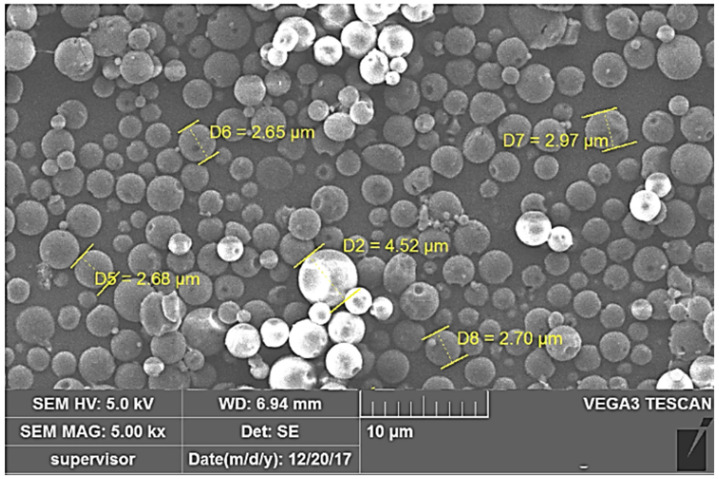
Surface morphology of the optimized formula of LVX-loaded microspheres.

Besides particle size, the fate of inhaled particles is highly dependent on the shape and/or morphological properties of particles [25]. Many reports have demonstrated that the shape of microparticles potentially influences the initial contact and subsequent phagocytosis of particles by macrophages [26]. Generally, spherical shape particles are more phagocytosed than elongated/rode shaped particles [27]. The particle morphology of the optimized LVX-loaded microspheres is shown in Figure 2. It is obvious that LVX-loaded microspheres have a relatively smooth surface with spherical geometry. This spherical geometry is anticipated to facilitate the uptake of the optimized microsphere by alveolar macrophages. In addition, no signs of aggregation were observed among the formulated microspheres, which could aid the efficient aerosolization of particles as DPIs.

### 2.3. Micromeritic Characteristics of LVX-Loaded Microspheres

The micromeritic characteristics of dry powder inhaler (DPI), including bulk density, powder flowability, moisture content, particle size aerodynamics, etc., significantly contribute to the quality and therapeutic efficacy of inhalation [28].

#### 2.3.1. Moisture Content

Moisture content significantly influences the flowability and dispersion behavior of DPI [29]. Powder flowability is adversely compromised in the presence of free water (moisture) in DPIs, which, in turn, detrimentally affects the aerosolization performance [30]. In the present study, Karl Fischer volumetric titration technique was employed to determine the moisture content in optimized LVX-loaded microspheres and it was 3.96 ± 0.41%, which is considered suitable for microsphere DPI [31].

#### 2.3.2. Flow Properties

Good flow property is essential for handling the dry-powder inhaler (DPI) comfortably; where good followability would ensure the delivery of an accurate dose, allow fluidization, and easy delivery of drug powder from the delivery system. As summarized in Table 2, the average bulk density of optimized LVX-loaded microspheres was less than 0.4 g/cm^3^, indicating that the microspheres are suitable to be easily dispersed upon aerosolization [32]. In addition, the results of flow properties of the optimized microsphere formulation, as evaluated in terms of angle of repose, Carr’s index and Hausner’s ratio, implied that the microsphere powder had a relatively good flowability, as compared with free drug. These findings suggested that microspheres had the potential to increase fine drug particle flowability and hence could aid in the consistent loading of fine drug particles into capsules and inhalation devices.

#### 2.3.3. Aerodynamic Diameter

Generally, particles having an aerodynamic diameter of 1 to 5 μm can readily accumulate in the deep region of lung [12]. In vitro deposition parameters of plain LVX and LVX-loaded microspheres are listed in Table 3. The mass median aerodynamic diameter (MMAD) of optimized LVX-loaded microspheres was 2.13 ± 1.24 µm, which was much less than that of free LVX (4.24 ± 1.37 µm) (Table 3). Additionally, LVX-loaded microspheres had a considerably greater fine particle fraction (FPF) than plain LVX. LVX-loaded microspheres had an FPF of 75.35 ± 1.42%, while plain medicine had an FPF of 53.70 ± 1.76%. These findings suggest that LVX-loaded microspheres have better aerosolization capabilities than plain LVX. Of note, LVX-loaded microspheres were primarily retained in the cascade impactor at lower stages (stages 5 and 6), which had smaller aerodynamic cut-off diameters (Table S1), because of their smaller MMAD and greater FPF. Collectively, these findings support the deep deposition of LVX-loaded microspheres into lung, compared to free LVX.

### 2.4. Structural Characteristics 

#### 2.4.1. Differential Scanning Calorimetry (DSC)

**Figure 3:**
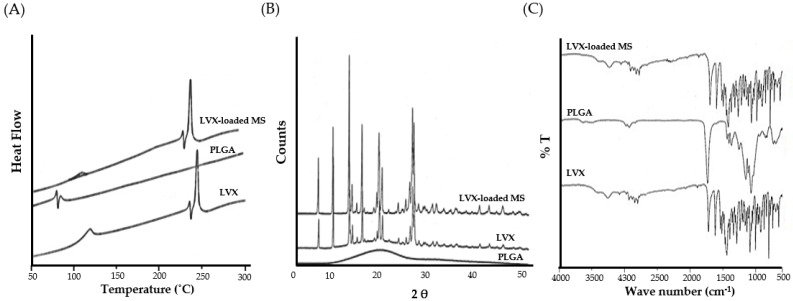
(**A**) DSC thermograms; (**B**) X-ray diffractograms; (**C**) FTIR spectra Pure LVX, PLGA polymer, and LVX-loaded microspheres.

To investigate the melting and crystallization behavior of pure LVX, PLGA, and LVX-loaded microspheres, DSC analysis was conducted (Figure 3A). The DSC results revealed that levofloxacin had a distinct endothermic peak at 243.15 °C, corresponding to the melting point of pure LVX. PLGA displayed an endothermic peak at 57.58 °C, corresponding to its glass transition temperature [33]. Of interest, the thermal profile of the optimized LVX-loaded microspheres retained the characteristic endothermic peak of levofloxacin, which indicated that LVX retained its crystalline nature upon entrapment within microspheres.

#### 2.4.2. X-ray Diffraction (XRD) Analysis 

Powder X-ray diffraction is a commonly used analytical technique used for gaining information about material crystallinity. X-ray diffractograms of pure levofloxacin, PLGA, and LVX-loaded microspheres are depicted in Figure 3B. The XRD diffractogram of pure levofloxacin showed a number of distinct sharp peaks at the 2θ values of 6.59°, 9.63°, 13.05°, 19.34°, and 26.36°, suggesting a high degree of crystallinity. On the other hand, the lack of sharp peaks in the diffractogram of PLGA confirms its amorphous nature. Interestingly, no obvious alterations were noted in the intensity or the number of peaks in LVX-loaded microspheres, compared to those of pure LVX. These findings nullify the existence of any interactions between LVX and PLGA in the formulated microspheres.

#### 2.4.3. Fourier Transform Infra-Red (FTIR) Spectroscopy

In order to scrutinize the possible drug–polymer interactions between levofloxacin and PLGA in LVX-loaded microspheres, FTIR analysis was conducted (Figure 3C). The FTIR Spectrum of pure levofloxacin exhibited sharp peaks at 3262 cm^−1^ for COOH (stretch), 1723 cm^−1^ for C=O (stretch), 1321 cm^−1^ for CH_3_ (stretch), and 1292 cm^−1^ for C-N (stretch). FTIR spectra of PLGA polymer showed intense band at 1747 cm^–1^, which was attributed to the stretching vibration of the carbonyl groups. Of interest, the FTIR spectrum of LVX-loaded microspheres retained all the characteristics absorption peaks of both pure levofloxacin and PLGA with no additional bands, suggesting the absence of any interactions between PLGA and levofloxacin. 

### 2.5. In Vitro Drug Release

**Figure 4:**
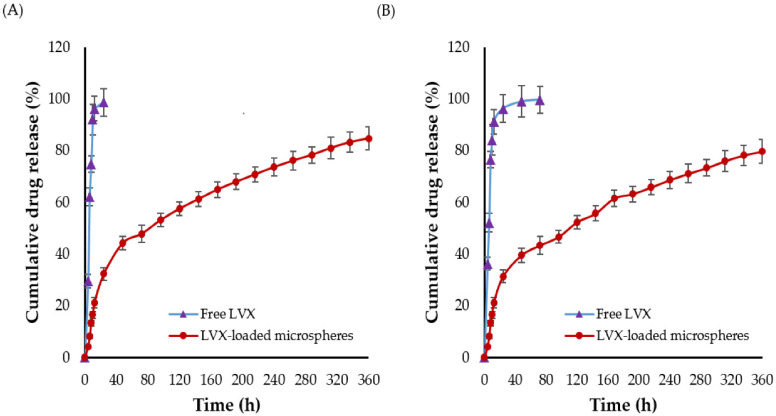
In vitro release of free LVX and LVX-loaded microspheres in (**A**) Acetate buffer (pH 4.4) and (**B**) Phosphate buffer (pH 7.4).

The in vitro dissolution/release profiles of both plain levofloxacin and LVX-loaded microspheres are shown in Figure 4. As shown in Figure 4, more than 75% of plain levofloxacin was released in both dissolution media within 8 h. The higher dissolution of plain levofloxacin in acetate buffer (pH 4.4), compared to phosphate buffer (pH 7.4), might be ascribed to the higher solubility of levofloxacin at acidic pH [34]. Interestingly, compared to plain levofloxacin, only 13% of the drug was released from LVX-loaded microspheres in 8 h. In addition, LVX-loaded microspheres exhibited a biphasic release pattern from drug-loaded microspheres (Figure 4), with a rapid burst release (~21%) in the first 12 h, followed by a sustained drug release for up to 15 d. The initial burst drug release from LVX-loaded microsphere might be attributed to the release of drug molecules located at the outer layers of microspheres [35]. Whilst the relatively delayed drug release from the microspheres might be ascribed to the slower drug diffusion from the core of microspheres and/or the slow erosion/degradation of polymeric matrix. Of note, there was no significant change in the in vitro release pattern of LVX from microspheres at any of the tested pH values.

### 2.6. Cytotoxicity of LVX-Loaded PLGA Microspheres against Alveolar Macrophages

**Figure 5:**
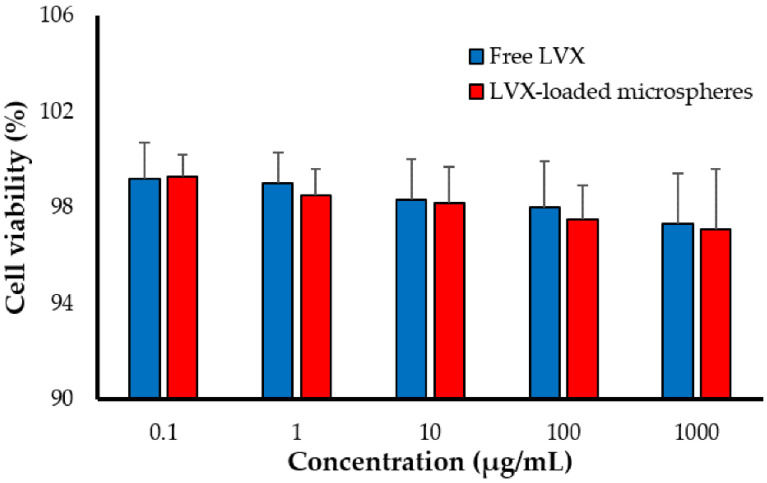
In vitro cell viability of A549 human alveolar basal epithelial cells upon treatment with LVX-loaded PLGA microspheres.

PLGA represents one of the most commonly used synthetic polymers for pharmaceutical applications. It has been approved by the Food and Drug Administration (FDA) for parenteral delivery [18]. However, its safety profile as an excipient for lung delivery has not been fully settled. Accordingly, the cytotoxic potential of LVX-loaded PLGA microspheres was assessed against adenocarcinomic human alveolar basal epithelial cells (A549). The toxicity of the free LVX and LVX-loaded microspheres was evaluated using tenfold dilutions (1000, 100, 10, 1, and 0.1 μg/mL). At all the tested concentrations, no significant difference in cell viability was observed between free drug and drug-loaded PLGA microspheres (Figure 5). These results suggest the safety/tolerability of PLGA microspheres, since there was no additional toxicity imposed on A549 cells by LVX-loaded PLGA microspheres compared to free LVX.

### 2.7. Stabilty Studies 

The storage stability of the formulated LVX-loaded microspheres is a critical element that influences the physicochemical properties of dry powder microparticles intended for inhalation such as physical appearance, particle size, entrapment efficiency, and drug loading. The storage stability results of optimized LVX-loaded microspheres are summarized in Table 4. All the investigated parameters did not show any remarkable changes during the six-month storage period at either refrigeration or accelerated temperatures (*p* > 0.05). These results suggest the long-term stability of the formulated optimized LVX-loaded microspheres at different storage conditions.

### 2.8. In Vivo Studies

#### 2.8.1. Pulmonary Pharmacokinetics

**Figure 6:**
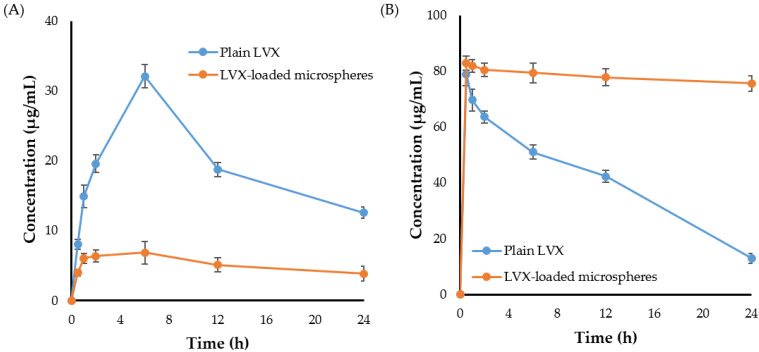
Mean drug concentration-time profile of plain LVX and LVX-loaded microspheres in (**A**) plasma and (**B**) lung following pulmonary inhalation.

The in vivo pharmacokinetic profile of LVX was studied following pulmonary administration of either plain drug or drug-loaded microspheres. Figure 6 represents the concentration-time curves of LVX in plasma and lung post inhalation of either plain LVX or LVX-loaded microspheres. As depicted in Figure 6A, compared to LVX-loaded microspheres, the blood concentration of plain LVX increased rapidly following drug inhalation reaching a significantly higher peak shortly after drug inhalation. Such relatively higher plasma drug concentration of plain drug following pulmonary administration might be attributed to the rapid drug absorption by lung alveoli enriched with abundant blood capillaries [36]. By contrary, the slower drug release from the microspheres, which would retard the availability of entrapped drug for absorption, might account for the relatively lower LVX plasma concentration following the inhalation of drug-loaded microspheres. 

The lung concentration-time curve of LVX following pulmonary administration of both plain LVX and LVX-loaded microspheres was shown in Figure 6B. It was obvious that higher lung drug concentrations were detected shortly following administration of both plain LVX and LVX-loaded microspheres. Most importantly, LVX-loaded microspheres showed the potential to maintain high drug concentration and to prolong LVX residence time within the lung for up to 24 h post microspheres inhalation. By contrary, a considerable decrease in plain LVX concentrations in the lung was detected shortly following drug inhalation. The rapid clearance of plain LVX from the pleural cavity to systemic circulation, as confirmed by a synchronized elevation in plasma drug level, might account for the drop in free drug concentration in the lung.

The pharmacokinetic parameters of LVX were determined and evaluated from lung tissue samples. As summarized in Table 5, LVX-loaded microspheres exerted longer half-life (t_1/2_) and mean residence time (MRT), compared to plain LVX. The MRT of plain LVX and LVX-loaded microspheres were 13.33 ± 0.87 h and 359.14 ± 20.88 h, respectively. These results might explain the prolonged effect of LVX-loaded microspheres due to the slow release of LVX from the drug-loaded microspheres. In addition, LVX-loaded microspheres exerted a remarkably higher AUC_0–24h_ in lung than plain drug. The AUC_0–24h_ values for LVX-loaded microspheres and plain LVX were 964.04 ±14.84 and 1854.08 ± 23.62 μg/mL.h, respectively. The increased AUC_0–24h_ of LVX-loaded microspheres might be ascribed to the longer retention and lower alveolar clearance of LVX-loaded microparticles from lung tissue when compared to free LVX. Our results are in consistent with that of Yang et al. [37] who demonstrated that entrapment of the phosphodiesterase-5 inhibitor, tadalafil, within PLGA microspheres could efficiently prolong the drug residence time in the lungs of Wistar rats following intra-tracheal administration, compared to tadalafil solution.

#### 2.8.2. Biodistribution Study

The success of targeted drug therapy is determined by adequate drug retention and efficient drug distribution within the site of action. Accordingly, the tissue distribution of plain LVX and LVX-loaded microspheres was traced following pulmonary administration. As summarized in Table 6, both plain LVX and LVX-loaded microspheres were readily and preferentially accumulated in the lung following pulmonary administration, with 78.95 ± 2.97% and 85.94 ± 3.61% of inhaled dose accumulated in the lung within 30 min post administration. Nonetheless, in the case of plain LVX, there was a steady decrease in drug lung accumulation over time. The accumulation level of plain LVX dropped from 78.95 ± 2.97% at 30 min to 28.60 ± 1.79 at 24 h post inhalation. On the other hand, LVX-loaded microspheres maintained higher levels of LVX in lung tissues for an extended period; ~75% of the inhaled dose was available within lung tissue even at 24 h post microsphere inhalation. In addition, the accumulation levels of LVX from microspheres in each tissue (spleen, liver or kidney) were significantly lower than those observed following plain LVX inhalation, demonstrating the efficacy of microspheres pulmonary delivery in alleviating drug distribution in tissues other than the lung. Collectively, these findings emphasize the efficacy of microspheres to maintain the pharmacological activity of entrapped drug (LVX), most likely by limiting its alveolar clearance from the lungs and prolonging drug residence time, which, in turn, would enable the efficient release of adequate drug concentrations within the site of action.

So far, the inhalation therapy was the mainstay treatment for many lung pathologies such as asthma, chronic obstructive pulmonary disease (COPD), and cancers [38,39]. Nevertheless, delivering drugs by inhalation is mainly restricted by the inherent defense mechanisms of the respiratory tract that expel inhaled drug particles out of the lungs and/or inactivate them once they have been deposited [12]. Accordingly, many strategies have been adopted to overcome the challenges/barriers of pulmonary drug delivery. Polymeric microparticles/nanoparticles have been evolved as promising delivery vehicles for pulmonary administration [40]. They showed the potential to offer high concentrations of entrapped drug and prolonged drug residence time in the lung while minimizing drug exposure to the blood circulation [41]. In the current study, microspheres were challenged as a delivery vehicle for the anti-tubercular fluoroquinolone levofloxacin (LVX), to the lung. Experimental design software (CCD) was adopted for fine tuning physicochemical characteristics of the formulated microspheres to be eligible for pulmonary administration. The obtained optimized LVX-loaded microsphere showed a MMAD of 2.13 ± 1.24 μm and a FPF of 75.35 ± 1.42, indicating good aerosolization properties (Table 3), which could allow deep deposition of inhaled microparticles into the alveolar space. In addition, no obvious toxicity of the microspheres was detected against lung alveolar basal epithelial cells (Figure 5), suggesting the safety of microspheres following pulmonary administration. Most importantly, compared to plain drug, LVX entrapment within microspheres extended LVX release for 2 weeks (Figure 4), which could account for the prolonged drug residence time in the lung (Table 4), and the minimized drug exposure to the blood circulation (Table 5). Overall, PLGA microspheres may be a viable carrier for combating pulmonary tuberculosis.

## 3. Materials and Methods

### 3.1. Materials

Levofloxacin (LVX) was generously provided from Micro labs limited (Bangaluru, India). Poly (lactic-coglycolic acid) (PLGA) was purchased from Loba chemicals (Mumbai, India). Dulbecco’s modified eagle medium (DMEM) was purchased from Hi-media (Mumbai, India). Fetal bovine serum (FBS) was obtained from Thermo Fisher Scientific (Waltham, MA, USA). 3-(4, 5-dimethylthaizole-2-yl)-2,5-diphenyl tetrazolium bromide (MTT) was procured from Sigma-Aldrich (St. Louis, MO, USA). All solvents and other chemical were of analytical reagent grade.

### 3.2. Preparation and Optimization of LVX-Loaded PLGA Microspheres 

#### 3.2.1. Fabrication of LVX-Loaded Microspheres 

Solvent vaporization technique was adopted to prepare LVX-loaded PLGA microspheres in the form of dry powder [42]. Briefly, 60 mg of LVX and 60 mg of PLGA polymer were dissolved in 6 mL dichloromethane to form the organic phase. The resultant organic phase was dispersed dropwise in 12 mL of aqueous phase containing 2% poly vinyl Alcohol (PVA). The mixture was stirred using high speed Polytron Homogenizer PT 6000 (Triad Scientific, Inc., Manasquan, NJ, USA) at a speed of 4000 rpm for 2 h to produce emulsion. Stirring at 4000 rpm was continued overnight to vaporize the organic solvent. The resulted microspheres were collected, washed trice with sterilized double distilled and freeze-dried until further use.

#### 3.2.2. Experimental Design

A 2-factor, 2-level central composite design was employed, using Stat-Ease^®^ Design Expert software, for the formulation of LVX-loaded microspheres and to explore the effect of definite formulation parameters on specific product characteristics. Drug amount (X_1_) and polymer content (X_2_) were selected as independent formulation variables, while particle size (Y_1_), drug content (Y_2_), and entrapment efficiency (Y_3_) were selected as formulation dependent responses. A total of 13 runs were carried out (Table 1) and Design Expert software was utilized to create a mathematical model for each dependent variable and the subsequent statistical analysis.

### 3.3. Structural Characterization of LVX-Loaded Microspheres

#### 3.3.1. Differential Scanning Calorimetry (DSC) Studies 

The DSC study was conducted to characterize the physical state of pure LVX, PLGA, and LVX in physical mixture with PLGA and LVX-loaded in microspheres. Thermograms were recorded using (DSC 60 Shimadzu, Tokyo, Japan) at temperatures ranging from 10–300 °C under a nitrogen atmosphere and a heating rate of 10 °C.min^−1^ [43].

#### 3.3.2. Powder X-ray Diffraction Analysis

The solid state form of pure LVX, PLGA, and LVX-loaded microspheres was characterized in the interval 2θ = 0–90° using D8 Advance Eco Powder X-ray diffractometer (Bruker AXS GmbH, Karlsruhe, Germany) supplemented with a 1D Vantec position sensitive detector.

#### 3.3.3. Fourier Transform Infra-Red Spectroscopy (FTIR) Study 

FTIR spectroscopy (PerkinElmer, Mississippi, MA, USA) was utilized to explore the possible chemical interactions between LVX and PLGA. The FTIR spectra, in the 4000 cm^−1^ –400 cm^−1^ wave number range, of Pure LVX, PLGA, and LVX-loaded microspheres were recorded as described previously [44].

### 3.4. Characterization of LVX-Loaded Microspheres 

#### 3.4.1. Particle Size Determination

Malvern nano ZS 90 zeta sizer (Malvern instrument, Worcestershire, UK) was used to estimate the particle size and polydispersity index, based on the principle of dynamic light scattering technique. 100 μL samples were diluted to 1 mL with Milli-Q water and particle size determinations were conducted at 25 °C [45].

#### 3.4.2. Microsphere Surface Morphology 

Surface morphologies of the optimized microsphere formulation were conducted using tungsten thermionic emission scanning electron microscopy (SEM; Tescan, Vega 35BH, Brno, Czech Republic). 

#### 3.4.3. Determination of Drug Loading and Encapsulation Efficiency

A definite weight of LVX-loaded microspheres (50 mg) was dissolved in 40 mL methanol, and subjected for sonication for 30 min using Vortex sonicator. The drug solution was then filtered. High performance liquid chromatography (HPLC, Shimadzu, Kyoto, Japan) was used to quantify the entrapped LVX concentration. Briefly, Chromatographic separation was carried out using Kromasil C18 column (250 × 4.6 mmm, 5 µm). The mobile phase consisted of acetonitrile: phosphate buffer solution (45:55 *v*/*v*, pH 4.4). Before quantification, the column was equilibrated at 40 °C for 1 h using mobile phase. The flow rate was 1 mL/min, and the UV detection was set at 294 nm. Levofloxacin was eluted at 6.13 min. The concentration of LVX was determined using a pre-established standard curve of LVX at different concentrations.

The actual drug loading and drug encapsulation efficiency were calculated using the following equations:

$$\mathrm{Drug}\;\mathrm{loading}\;\left(\%\right)=\frac{\mathrm{Weight}\;\mathrm{of}\;\mathrm{drug}\;\mathrm{in}\;\mathrm{microspheres}}{\mathrm{Weight}\;\mathrm{of}\;\mathrm{microspheres}}\times 100$$

$$\mathrm{Encapsulation}\;\mathrm{efficiency}\;\left(\%\right)=\frac{\mathrm{Drug}\;\mathrm{conent}\;\mathrm{in}\;\mathrm{microspheres}}{\mathrm{Initial}\;\mathrm{drug}\;\mathrm{content}}\times 100$$

### 3.5. Micrometric Properties of Prepared Microspheres

#### 3.5.1. Determination of Bulk Density (ρ_b_)

A 10 mL tared graduated cylinder filled with drug-loaded microspheres was used to estimate the bulk density of the samples. After direct volume and mass measurement, the bulk density was calculated using the following equation:

$$\mathrm{Bulk}\;\mathrm{density}\;\left(\rho_{b}\right)=\frac{\mathrm{Mass}}{\mathrm{Volume}}$$

#### 3.5.2. Flowability Testing

Hausner’s ratio and Carr’s index were calculated as indicators of the flow properties of prepared drug-loaded microspheres.

(1) $$\mathrm{Hausner}’s\;\mathrm{ratio}=\frac{\rho_{t}}{\rho_{b}}$$

(2) $$\mathrm{Carr}’s\;\mathrm{index}=\frac{\rho_{t}-\rho_{b}}{\rho_{t}}\times 100$$

where ρ_b_ and ρ_t_ are bulk density and tapped density of the microspheres, respectively. 

The angle of repose was also estimated by passing the microspheres through a funnel placed on a horizontal surface [46]. The angle of repose was calculated from the ratio of the height to the base radius of the heap formed.

#### 3.5.3. Determination of Moisture Content

The Karl Fischer volumetric titration method was implemented to determine the moisture content in the formulated microspheres [47]. The Karl Fischer Auto Titrator contains a titration cell placed in a platinum electrode and a port to introduce the known amount of sample and Karl Fischer reagent. A definite weight of LVX-loaded microparticles was dissolved in dry methanol and placed in titration cell and the electrical conductivity was measured potentiometrically using platinum electrode. The alteration in electrical conductivity accomplished by drug-loaded microspheres was balanced by adding measured volumes of Karl Fischer reagent. The moisture content was obtained by determining the amount of reagent required to restore the electrical conductivity to its pre-microspheres value. 

#### 3.5.4. In Vitro Deposition by Andersen Cascade Impactor

The Andersen cascade impactor is an equipment that is commonly utilized for studying the aerodynamic characteristics of inhaled DPIs in vitro. For the determination of the aerodynamic diameter of the formulated LVX-loaded microspheres, seven capsules were filled individually with 50 mg of drug-loaded microspheres. The filled capsules were then inserted into an inhaler device connected to the cascade impactor and drug-loaded microspheres were delivered to the impactor at a rate of 27.8 L/min for 10 sec. HPLC system was used to analyze drug particle deposited on each stage of cascade impactor. In addition, to characterize the deposition profile of the prepared LVX-loaded microspheres, fine particle dose (FPD), fine particle fraction (FPF), and emitted dose (ED) were determined using the following equations:

Fine particle dose=mass of fine particles on stage 2 through 7

$$\mathrm{Fine}\;\mathrm{particle}\;\mathrm{fraction}\;\left(\mathrm{FPF}\right)=\frac{\mathrm{Fine}\;\mathrm{particle}\;\mathrm{dose}}{\mathrm{Initial}\;\mathrm{particle}\;\mathrm{mass}}\times 100$$

$$\mathrm{Emitted}\;\mathrm{dose}\;\left(\mathrm{ED}\right)=\frac{\mathrm{Total}\;\mathrm{particle}\;\mathrm{mass}\;\mathrm{on}\;\mathrm{all}\;\mathrm{stages}}{\mathrm{Initial}\;\mathrm{particle}\;\mathrm{mass}}\times 100$$

Finally, an online MMAD calculator was used to calculate the mass median aerodynamic diameter (MMAD) and geometric standard deviation (GSD).

### 3.6. In Vitro Studies on Drug Release of the Optimized Microspheres

In vitro release of levofloxacin from developed LVX-loaded microspheres was monitored at two different pH values; acetate buffer (pH 4.4) and phosphate buffer (pH 7.4) to simulate the intracellular environment of phagosomes and lysosomes in the lung. In brief, 25 mg of LVX-loaded microspheres was suspended in 500 mL of dissolution medium maintained at 37 ± 0.5 °C at a rotation speed of 100 rpm. At regular time intervals (1, 2, 4, 8, 12, and 24 h), 1 mL aliquot samples were withdrawn and analyzed by HPLC to determine drug concentration. The initial volume of dissolution medium was maintained by replenishing equal volume of fresh dissolution media after each withdrawal. 

### 3.7. In Vitro Cytotoxicity of LVX-Loaded PLGA Microspheres against Alveolar Murine Macrophages

Human adenocarcinomic alveolar basal epithelial cells (A549) were grown in Dulbecco’s modified eagle medium (DMEM) supplement with 10% FBS in a humidified atmosphere with 5% CO_2_ at 37 °C. In vitro cytotoxicity of free LVX or LVX-loaded microspheres on A549 cells was estimated using the standard MTT assay. Briefly, 1 × 10^5^ cells were plated in a 96-well plate and were allowed to adhere in a standard culture environment. At 24 h post-incubation, spent media were replaced with fresh media containing serial dilutions (0.1–1000 μg/mL) of free LVX or LVX-loaded microspheres and the cells were further incubated for 24 h. The cells were then washed trice with cold phosphate buffer saline, and 100 μL of MTT solution (0.5 mg/mL) was added. After 4 h incubation, the MTT reagent was aspirated and 200 μL of dimethyl sulfoxide (DMSO) was added to each well to dissolve the formed formazan crystals before recording the absorbance at 570 nm using an ELISA microplate reader (ELX-800 BioTek, Midland, ON, Canada). Untreated cells served as control.

### 3.8. Stability Studies

The optimized LVX-loaded microspheres were subjected to stability studies for six months. The stability protocol was designed in accordance with ICH guidelines. LVX-loaded microspheres were filled into capsules, kept in glass vials and placed in a stability chamber under different storage conditions: 4 ± 1 °C/ambient RH, 25 ± 2 °C/60 ± 5% RH, and 40 ± 2 °C/75 ± 5% RH. The visual appearance, particle size, encapsulation efficiency and drug loading were periodically investigated for up to 6 m.

### 3.9. In Vivo Studies

#### 3.9.1. Animals

A six weeks old male Swiss albino mice (20–25 g) were procured from Biogen Laboratory Animal Facility (Bengaluru, India). The animals were housed under standard environmental conditions of temperature (25 ± 1 °C) and humidity (50 ± 10%) and had free access for lab feed and water. The experimental protocol was reviewed and approved by the Institutional Animal Ethical Committee of JSS College of Pharmacy, Mysuru. (Approval Number P 1-321). 

#### 3.9.2. Pharmacokinetic and Organ Biodistribution Study of LVX-Loaded Microspheres

For pharmacokinetic study, Swiss albino mice were divided randomly into two groups; control group receiving free LVX and treatment group receiving LVX-loaded microspheres. For pulmonary administration of dry powder aerosols, a nose-only inhalation exposure apparatus was used [48,49]. The components and the validation of the home-made nose-only inhalation exposure apparatus were depicted in Figure S2. LVX dose was 10 mg/kg for all animals. At scheduled time points; 0.5, 1, 2, 6, 12, and 24 h (n = 5 per time), blood samples (200 μL) were collected from each mice into heparinized micro-tubes, and centrifuged at 5000× *g* rpm for 20 min to separate plasma. 100 μL of obtained plasma samples were then mixed with 100 μL of acetonitrile for 30 sec by vortex to extract LVX. The supernatants were separated by centrifugation (4000× *g* rpm, 5 min). Finally, the samples were filtrated through 0.22 µm membrane filter and drug concentration was quantified by HPLC system. Pharmacokinetic parameters of LVX, including peak plasma concentration (C_max_), half-life (t_1/2_), area under the concentration–time curve (AUC_0–24h_) and median residence time (MRT), were calculated with a non-compartmental model using PKSolver 2.0 software.

For biodistribution study, following blood collection at definite time intervals, mice were euthanized, and lungs, liver, heart, kidney, and spleen were dissected. The dissected organs were rinsed with cold phosphate buffer saline (pH 7.4), dried, weighed, and homogenized at 10,000× *g* rpm for 1 min under an ice bath. To extract LVX, 100 μL of acetonitrile was mixed with 100 μL aliquots of each sample and vortexed for 5 min. After centrifugation (10,000× *g* rpm, 10 min), the supernatants were separated and analyzed for LVX using the HPLC system.

### 3.10. Statistical Analysis

The data was shown as mean ± SD. The unpaired Student’s *t*-test (SPSS software version 16; SPSS Inc., Chicago, IL, USA) was used for statistical significance analysis. A *p* < 0.05 showed that there was a significant difference. 

## 4. Conclusions

In this study, inhalable microspheres of the anti-TB fluoroquinolone, levofloxacin, were prepared using solvent vaporization technique. Central composite design was adopted to obtain an optimized formula eligible for pulmonary administration. Micromeritic studies confirmed the adequacy of optimized LVX-loaded microspheres for pulmonary inhalation. In vitro cytotoxicity study confirmed the safety of PLGA microspheres against lung cells. In addition, the inhalable microspheres showed better efficacy than plain drug in terms of prolonging drug residence in the lung. The longer drug residence time along with prolonged drug release at the infected site from the formulated inhalable microspheres justified their potential to efficiently alleviate TB, while reducing dosing frequency and lessening drug-related systemic side effects. Collectively, the prepared LVX-loaded PLGA microspheres might be considered as promising candidates for the effective treatment of TB with a higher patient treatment adherence. Nevertheless, chronic toxicological data on inhalable PLGA particles has to be fully elucidated prior to their adoption in clinical settings.

## Figures and Tables

**Table 1:** Experimental design matrix of the central composite design with experimental results.

Formula	Coded Values		Actual Values of Independence Values		Responses Variables		
	X_1_	X_2_	X_1_ (mg)	X_2_ (mg)	Y_1_ (µm)	Y_2_ (%)	Y_3_ (%)
MS1	1.414	0	11.03	7.5	3.8 ± 0.45	32.9 ± 2.1	64.87 ± 4.8
MS2	0	1.414	7.5	11.03	3.2 ± 0.37	41.98 ± 3.2	71.87 ± 6.5
MS3	+1	+1	10	10	2.9 ± 0.31	39.97 ± 2.9	75.98 ± 6.3
MS4	−1	−1	5	5	2.9 ± 0.30	39.09 ± 2.7	73.27 ± 5.9
MS5	0	−1.414	7.5	3.96	4.2 ± 0.29	36.98 ± 2.5	76.59 ± 6.2
MS6	0	0	7.5	7.5	2.9 ± 0.19	38.87 ± 3.1	78.25 ± 6.1
MS7	−1	+1	5	10	2.4 ± 0.17	42.03 ± 2.9	74.78 ± 5.8
MS8	0	0	7.5	7.5	2.9 ± 0.20	39.09 ± 2.6	80.06 ± 6.8
MS9	0	0	7.5	7.5	2.8 ± 0.19	39.65 ± 2.6	79.98 ± 6.5
MS10	0	0	7.5	7.5	2.9 ± 0.19	38.93 ± 2.4	81.26 ± 5.9
MS11	0	0	7.5	7.5	3.1 ± 0.21	38.76 ± 3.5	80.01 ± 5.7
MS12	−1.414	0	3.96	7.5	3.2 ± 0.18	34.09 ± 1.8	60.87 ± 4.7
MS13	+1	−1	10	5	3.6 ± 0.22	31.09 ± 1.9	69.65 ± 4.5

X_1_: levofloxacin concentration; X_2_: PLGA concentration; Y_1_: particle size; Y_2_: percentage drug loading; and Y_3_: percentage entrapment efficiency.

**Table 2:** Micromeritic properties of free LVX and optimized LVX-loaded microspheres.

Parameters	Pure LVX	LVX-Loaded Microspheres
Bulk Density	0.22 ± 0.02 g/cm^3^	0.39 ± 0.06 g/cm^3^
Tapped Density	0.31 ± 0.01 g/cm^3^	0.48 ± 0.08 g/cm^3^
Carr’s Index	29.03	18.75
Hausner’s ratio	1.41	1.23
Angle of Repose (θ)	42 ± 2°	29 ± 1°

**Table 3:** Aerodynamic parameters of free LVX and optimized LVX-loaded microspheres.

Parameters	Pure LVX	LVX-Loaded Microspheres
Recovered dose (RD (µg))	99.05 ± 0.99	91.76 ± 1.54
Emitted dose (ED (µg))	82.34 ± 1.99	83.12 ± 2.18
Fine particle dose (FPD (µg))	51.87 ± 1.23	67.98 ± 1.54
Fine particle fraction (FPF (%))	53.70 ± 1.76	75.35 ± 1.42
Mass median aerodynamic diameter (MMAD (µm))	4.24± 1.37	2.13 ± 1.24
Geometric standard deviation (GSD)	2.38 ± 1.09	3.61 ± 0.87

Data represent mean ± SD of three independent experiments.

**Table 4:** Stability study of LVX-loaded microspheres upon long-term storage at different storage conditions.

Stability Condition	Time Point	Drug Loading (%)	Particle Size (μm)	Entrapment Efficiency (%)
4 ± 1 °C ambient RH	Initial	40.85 ± 2.09	2.86 ± 0.26	77.80 ± 1.98
	2 weeks	40.93 ± 2.13	2.87 ± 0.21	77.45 ± 1.56
	1 month	40.68 ± 1.98	2.89 ± 0.24	77.76 ± 1.83
	2 months	40.81 ± 1.09	2.93 ± 0.29	77.79 ± 1.11
	3 months	40.34 ± 1.11	2.96 ± 0.26	77.82 ± 1.35
	6 months	40.69 ± 2.01	2.99 ± 0.29	77.59 ± 1.55
25 ± 2 °C 60 ± 5% RH	Initial	40.85 ± 1.10	2.86 ± 0.26	77.80 ± 1.86
	2 weeks	40.67 ± 0.99	2.89 ± 0.21	77.91 ± 2.01
	1 month	40.09 ± 1.56	2.91 ± 0.24	77.39 ± 1.72
	2 months	40.63 ± 2.04	2.95 ± 0.29	77.69 ± 1.20
	3 months	40.77 ± 1.87	2.97 ± 0.26	77.79 ± 1.71
	6 months	40.81 ± 1.43	3.01 ± 0.29	77.81 ± 1.84
40 ± 2 °C 75 ± 5% RH	Initial	40.85 ± 1.25	2.86 ± 0.26	77.80 ± 1.77
	2 weeks	39.97 ± 1.22	2.90 ± 0.31	77.79 ± 2.00
	1 month	39.09 ± 1.89	2.92 ± 0.28	77.32 ± 2.14
	2 months	38.12 ± 1.59	2.98 ± 0.30	76.97 ± 0.99
	3 months	37.64 ± 1.45	3.01 ± 0.22	76.01 ± 1.54
	6 months	33.87 ± 1.37	3.03 ± 0.34	73.89 ± 1.64

Data represents mean ± SD of three independent determinations.

**Table 5:** Non-compartmental pharmacokinetic parameters of plain LVX and LVX-loaded microspheres following pulmonary administration.

Pharmacokinetic Parameters	Plasma		Lung	
	Plain LVX	LVX-Loaded Microspheres	Plain LVX	LVX-Loaded Microspheres
C_max_ (μg/mL)	32.08 ± 2.6	6.84 ± 0.76	78.92 ± 4.50	82.86 ± 5.76
T_1/2_ (h)	14.03 ± 0.71	22.68 ± 1.98	9.72 ± 0.51	248.75 ± 10.12
AUC_0–24_ (μg/mL.h)	468.59 ± 13.81	125.74 ± 14.13	964.04 ±14.84	1854.08 ± 23.62
MRT (h)	22.34 ± 2.15	33.78 ± 2.81	13.33 ± 0.87	359.14 ± 20.88

Data represents mean ± SD. (n = 5).

**Table 6:** In vivo organ biodistribution after pulmonary administration of plain LVX and LVX-loaded microspheres.

Formulation	Organ	% Dose Detected					
		0.5 h	1 h	2 h	6 h	12 h	24 h
Plain LVX	Serum	9.70 ± 1.81	15.91 ± 2.18	18.80 ± 1.74	26.78 ± 2.51	17.65 ± 1.29	13.12 ± 1.11
	Lung	78.95 ± 2.97	69.76 ± 2.26	60.53 ± 2.32	49.12 ± 1.34	42.79 ± 1.53	28.60 ± 1.79
	Liver	2.37 ± 1.06	3.01 ± 0.97	4.27 ± 1.11	5.05 ± 1.72	7.07 ± 1.65	8.35 ± 1.96
	Spleen	1.38 ± 0.40	2.75 ± 0.67	4.35 ± 1.02	4.19 ± 1.11	4.72 ± 0.98	5.04 ± 1.14
	Kidney	ND *	0.62 ± 0.20	1.17 ± 0.45	2.04 ± 0.41	1.78 ± 0.34	1.49 ± 0.47
LVX-loaded microspheres	Serum	4.24 ± 1.45	5.8 ± 1.69	6.34 ± 1.74	6.525 ± 1.52	4.755 ± 1.49	3.735 ± 1.24
	Lung	85.94 ± 3.61	82.32 ± 2.11	80.15 ± 2.43	78.05 ± 1.42	76.79 ± 1.68	74.11 ± 2.05
	Liver	1.71 ± 0.64	2.15 ± 0.79	2.68 ± 0.83	2.98 ± 1.01	3.36 ± 1.12	3.17 ± 0.98
	Spleen	0.55 ± 0.11	0.72 ± 0.16	0.95 ± 0.14	1.3 ± 0.19	0.81 ± 0.21	1.18 ± 0.26
	Kidney	ND *	0.57 ± 0.19	1.29 ± 0.23	1.69 ± 0.31	1.59 ± 0.45	0.45 ± 0.11

All data represents the mean ± SD. * ND: not detectable. (n = 5).

## Data Availability

All data can be found within this article and its supplementary information.

## Supplementary Materials

The following supporting information can be downloaded at: https://www.mdpi.com/article/10.3390/ph15050560/s1, Figure S1: Particle size distribution of optimized LVX-loaded microspheres; Figure S2: “Nose-only” inhalation apparatus used for pulmonary administration of LVX-loaded microspheres to mice; Table S1: Levofloxacin deposition onto different stages of Anderson Cascade.

## References

[1] Smith I. (2003). *Mycobacterium tuberculosis* pathogenesis and molecular determinants of virulence. Clin. Microbiol. Rev..

[2] Gengenbacher M., Kaufmann S.H.E. (2012). *Mycobacterium tuberculosis*: Success through dormancy. FEMS Microbiol. Rev..

[3] Lee J.Y. (2015). Diagnosis and treatment of extrapulmonary tuberculosis. Tuberc. Respir. Dis..

[4] World Health Organization WHO Consolidated Guidelines on Tuberculosis 2021. https://apps.who.int/iris/bitstream/handle/10665/340255/9789240022676-eng.pdf.

[5] World Health Organization Updates on the Treatment of Drug-Susceptible Tuberculosis 2021. https://www.who.int/news/item/14-06-2021-who-announces-updates-on-the-treatment-of-drug-susceptible-tuberculosis_14062021.

[6] Richeldi L., Covi M., Ferrara G., Franco F., Vailati P., Meschiari E., Fabbri L.M., Velluti G. (2002). Clinical use of Levofloxacin in the long-term treatment of drug resistant tuberculosis. Monaldi Arch. Chest Dis..

[7] Pranger A.D., van der Werf T.S., Kosterink J.G.W., Alffenaar J.W.C. (2019). The Role of Fluoroquinolones in the Treatment of Tuberculosis in 2019. Drugs.

[8] Lanoix J.P., Chaisson R.E., Nuermberger E.L. (2016). Shortening Tuberculosis Treatment with Fluoroquinolones: Lost in Translation?. Clin. Infect. Dis..

[9] Aubry A., Pan X.S., Fisher L.M., Jarlier V., Cambau E. (2004). *Mycobacterium tuberculosis* DNA gyrase: Interaction with quinolones and correlation with antimycobacterial drug activity. Antimicrob. Agents Chemother..

[10] Johnson J.L., Hadad D.J., Boom W.H., Daley C.L., Peloquin C.A., Eisenach K.D., Jankus D.D., Debanne S.M., Charlebois E.D., Maciel E. (2006). Early and extended early bactericidal activity of levofloxacin, gatifloxacin and moxifloxacin in pulmonary tuberculosis. Int. J. Tuberc. Lung Dis..

[11] Campbell S., Smeets N., Jafar Mazumder M.A., Sheardown H., Al-Ahmed A. (2019). Drug Delivery: Localized and Systemic Therapeutic Strategies with Polymer Systems. Functional Polymers.

[12] Labiris N.R., Dolovich M.B. (2003). Pulmonary drug delivery. Part I: Physiological factors affecting therapeutic effectiveness of aerosolized medications. Br. J. Clin. Pharmacol..

[13] Murgia X., de Souza Carvalho C., Lehr C.-M. (2014). Overcoming the pulmonary barrier: New insights to improve the efficiency of inhaled therapeutics. Eur. J. Nanomed..

[14] Tse J.Y., Kadota K., Imakubo T., Uchiyama H., Tozuka Y. (2021). Enhancement of the extra-fine particle fraction of levofloxacin embedded in excipient matrix formulations for dry powder inhaler using response surface methodology. Eur. J. Pharm. Sci..

[15] Laohapojanart N., Ratanajamit C., Kawkitinarong K., Srichana T. (2021). Efficacy and safety of combined isoniazid-rifampicin-pyrazinamide-levofloxacin dry powder inhaler in treatment of pulmonary tuberculosis: A randomized controlled trial. Pulm. Pharmacol. Ther..

[16] Akdag Cayli Y., Sahin S., Buttini F., Balducci A.G., Montanari S., Vural I., Oner L. (2017). Dry powders for the inhalation of ciprofloxacin or levofloxacin combined with a mucolytic agent for cystic fibrosis patients. Drug Dev. Ind. Pharm..

[17] Mehta P., Bothiraja C., Kadam S., Pawar A. (2018). Potential of dry powder inhalers for tuberculosis therapy: Facts, fidelity and future. Artif. Cells Nanomed. Biotechnol..

[18] Emami F., Mostafavi Yazdi S.J., Na D.H. (2019). Poly(lactic acid)/poly(lactic-co-glycolic acid) particulate carriers for pulmonary drug delivery. J. Pharm. Investig..

[19] Coowanitwong I., Arya V., Kulvanich P., Hochhaus G. (2008). Slow release formulations of inhaled rifampin. AAPS J..

[20] Lu D., Garcia-Contreras L., Xu D., Kurtz S.L., Liu J., Braunstein M., McMurray D.N., Hickey A.J. (2007). Poly (lactide-co-glycolide) microspheres in respirable sizes enhance an in vitro T cell response to recombinant *Mycobacterium tuberculosis* antigen 85B. Pharm. Res..

[21] Rawat A., Majumder Q.H., Ahsan F. (2008). Inhalable large porous microspheres of low molecular weight heparin: In vitro and in vivo evaluation. J. Control. Release Off. J. Control. Release Soc..

[22] Thomas R.J. (2013). Particle size and pathogenicity in the respiratory tract. Virulence.

[23] Darquenne C. (2012). Aerosol deposition in health and disease. J. Aerosol. Med. Pulm. Drug Deliv..

[24] Champion Julie A., Mitragotri S. (2006). Role of target geometry in phagocytosis. Proc. Natl. Acad. Sci. USA.

[25] Lippmann M., Yeates D.B., Albert R.E. (1980). Deposition, Retention, and Clearance of Inhaled Particles. Br. J. Ind. Med..

[26] Geiser M. (2002). Morphological aspects of particle uptake by lung phagocytes. Microsc. Res. Tech..

[27] Kwon Y.B., Kang J.H., Han C.S., Kim D.W., Park C.W. (2020). The Effect of Particle Size and Surface Roughness of Spray-Dried Bosentan Microparticles on Aerodynamic Performance for Dry Powder Inhalation. Pharmaceutics.

[28] Shetty N., Cipolla D., Park H., Zhou Q.T. (2020). Physical stability of dry powder inhaler formulations. Expert Opin. Drug Deliv..

[29] Lu X.-Y., Chen L., Wu C.-Y., Chan H.-K., Freeman T. (2017). The Effects of Relative Humidity on the Flowability and Dispersion Performance of Lactose Mixtures. Materials.

[30] Murata S., Ito H., Izumi T., Chikushi A. (2006). Effect of the moisture content in aerosol on the spray performance of Stmerin D HFA preparations. Chem. Pharm. Bull..

[31] Singh D.J., Lohade A.A., Parmar J.J., Hegde D.D., Soni P., Samad A., Menon M.D. (2012). Development of Chitosan-based Dry Powder Inhalation System of Cisplatin for Lung Cancer. Indian J. Pharm. Sci..

[32] Chaurasiya B., Zhao Y.-Y. (2021). Dry Powder for Pulmonary Delivery: A Comprehensive Review. Pharmaceutics.

[33] Makadia H.K., Siegel S.J. (2011). Poly Lactic-co-Glycolic Acid (PLGA) as Biodegradable Controlled Drug Delivery Carrier. Polymers.

[34] Sabry H.S., Al-Shohani A.D.H., Mahmood S.Z. (2021). Formulation and Evaluation of Levofloxacin and Betamethasone Ophthalmic Emulgel. J. Pharm. Bioallied Sci..

[35] Denkbaş E.B., Seyyal M., Pişkin E. (1999). 5-fluorouracil loaded chitosan microspheres for chemoembolization. J. Microencapsul..

[36] Patton J.S., Fishburn C.S., Weers J.G. (2004). The Lungs as a Portal of Entry for Systemic Drug Delivery. Proc. Am. Thorac. Soc..

[37] Yang Z., Wang L., Tian L., Zhang X., Huang G. (2019). Tadalafil-loaded PLGA microspheres for pulmonary administration: Preparation and evaluation. Braz. J. Pharm. Sci..

[38] Kuzmov A., Minko T. (2015). Nanotechnology approaches for inhalation treatment of lung diseases. J. Control. Release.

[39] Borghardt J.M., Kloft C., Sharma A. (2018). Inhaled Therapy in Respiratory Disease: The Complex Interplay of Pulmonary Kinetic Processes. Can. Respir. J..

[40] Saigal A., Ng W.K., Tan R.B., Chan S.Y. (2015). Controlled Release Inhalable Polymeric Microspheres for Treatment of Pulmonary Arterial Hypertension. Curr. Pharm. Des..

[41] Harush-Frenkel O., Bivas-Benita M., Nassar T., Springer C., Sherman Y., Avital A., Altschuler Y., Borlak J., Benita S. (2010). A safety and tolerability study of differently-charged nanoparticles for local pulmonary drug delivery. Toxicol. Appl. Pharm..

[42] O’Donnell P.B., McGinity J.W. (1997). Preparation of microspheres by the solvent evaporation technique. Adv. Drug Deliv. Rev..

[43] Abu Lila A.S., Soliman M.S., Kiran H.C., Gangadharappa H.V., Younes K.M., Khafagy E.-S., Shehata T.M., Ibrahim M.M., Abdallah M.H. (2021). Tamoxifen-loaded functionalized graphene nanoribbons for breast cancer therapy. J. Drug Deliv. Sci. Technol..

[44] Hasan A.A., Samir R.M., Abu-Zaid S.S., Abu Lila A.S. (2020). Revitalizing the local anesthetic effect of Mebeverine hydrochloride via encapsulation within ethosomal vesicular system. Colloids Surf. B Biointerfaces.

[45] Al Saqr A., Khafagy E.-S., Alalaiwe A., Aldawsari M.F., Alshahrani S.M., Anwer M.K., Khan S., Lila A.S.A., Arab H.H., Hegazy W.A.H. (2021). Synthesis of Gold Nanoparticles by Using Green Machinery: Characterization and In Vitro Toxicity. Nanomaterials.

[46] Beakawi Al-Hashemi H.M., Baghabra Al-Amoudi O.S. (2018). A review on the angle of repose of granular materials. Powder Technol..

[47] Krasucka D.M., Kos K., Cybulski W.A., Mitura A., Łysiak E., Pietroń W.J. (2012). Karl Fisher determination of residual moisture in veterinary vaccines—Practical implementation in market monitoring. Acta Pol. Pharm..

[48] Sharma R., Saxena D., Dwivedi A.K., Misra A. (2001). Inhalable microparticles containing drug combinations to target alveolar macrophages for treatment of pulmonary tuberculosis. Pharm. Res..

[49] Kaur J., Muttil P., Verma R.K., Kumar K., Yadav A.B., Sharma R., Misra A. (2008). A hand-held apparatus for “nose-only” exposure of mice to inhalable microparticles as a dry powder inhalation targeting lung and airway macrophages. Eur. J. Pharm. Sci..

