# Supplementary material for: Pulmonary Targeting of Levofloxacin Using Microsphere-Based Dry Powder Inhalation
Source: Pharmaceuticals (Basel). 2022 Apr 30;15(5):560. doi: 10.3390/ph15050560 (PMC9145307; doi:10.3390/ph15050560)
Supplement: Supplementary file 1 [file pharmaceuticals-15-00560-s001.zip › pharmaceuticals-1667671-supplementary.pdf]

Supplementary Figure S1: Particle size distribution of optimized LVX-loaded microspheres

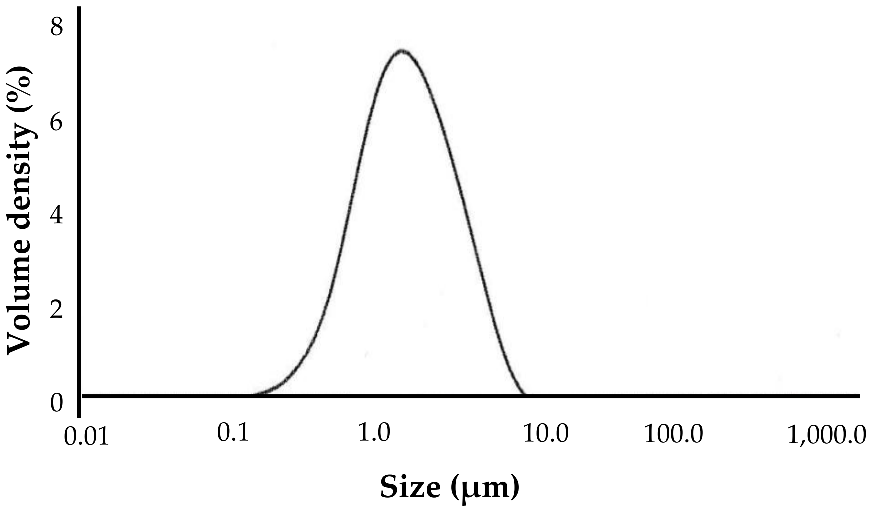

Supplementary Figure S2: "Nose-only" inhalation apparatus used for administering inhalable microparticles to mice.

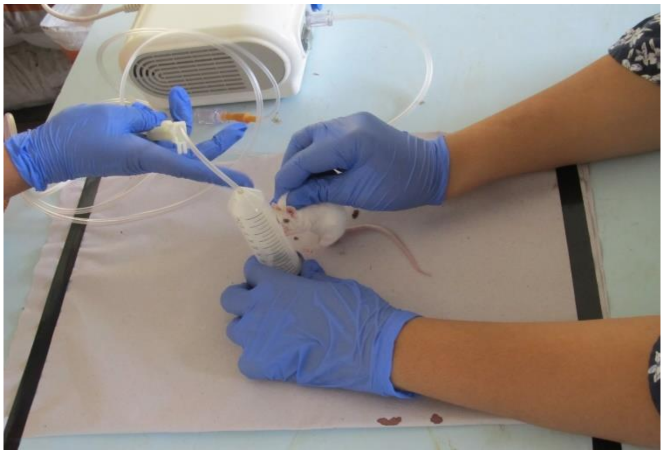

The delivery chamber consisted of a 50-ml plastic centrifuge tube with a hole of ~0.5 cm diameter at a distance of about 2.5 cm from the rim. The powder for inhalation was weighed in the cap. A length of tubing (i.d. ~ 2mm) was inserted into the tube from the apex of the taper (through another orifice) to a clearance of about 2.5–5mm from the inner surface of the cap. The tubing was connected to the air pump to admit a turbulent air stream, at a constant rate, for fluidizing the powder for 60 seconds. The home-made nose-only inhalation exposure apparatus was validated for delivering equal doses of microspheres as follows:

1- The nose-only inhalation exposure apparatus was primed with a few blank runs before using for in vivo experiment to nullify the possibility of administered dose loss by adsorption of aerosolized particles on the surface of the inhalation chamber.

2- After priming the inhalation apparatus, a constant weight of free drug or drug loading microspheres were charged into the inhalation apparatus.

3- A vacuum pump operating at low voltage was adopted to fluidize the powder bed in the plastic centrifuge tube (inhalation apparatus) for a constant period (60 sec.)

4- After the end of inhalation time, the remaining dose in the inhalation chamber was collected, weighed and the drug content was estimated.

Supplementary Table S1: Levofloxacin amount deposited into different stages of Anderson Cascade

Cascade impactor stages	Amount deposited (μg)	
	Pure LVX	LVX-loaded microspheres
Device	0.81 ± 0.04	2.16 ± 0.14
Capsule	2.5 ± 0.7	2.52 ± 0.17
Mouth piece	10.2 ± 1.10	3.03 ± 0.21
Induction port	10.8 ± 0.99	4.16 ± 0.51
Pre-separator	6.20 ± 0.45	3.50 ± 0.22
Stage 1	15.76 ± 1.12	9.38 ± 0.61
Stage 2	12.29 ± 1.07	8.50 ± 0.45
Stage 3	12.20 ± 0.98	11.10 ± 0.76
Stage 4	13.98 ± 1.31	12.21 ± 0.81
Stage 5	8.72 ± 0.75	11.10 ± 0.91
Stage 6	6.07 ± 0.34	15.63 ± 1.21
Stage 7	0.455 ± 0.01	17.13 ± 1.40
Stage filter	0.001 ± 0.00	0.001 ± 0.00
